# The Prevention of Heart Failure Risk: Assessment and Early Intervention along the Cardiovascular Continuum

**DOI:** 10.1007/s11897-026-00751-2

**Published:** 2026-03-19

**Authors:** Elisa Grossmann, Hannah-Lou Schilling, Christina Magnussen

**Affiliations:** 1 Department of Cardiology, University Heart and Vascular Center Hamburg, University Medical Center Hamburg-Eppendorf, Martinistraße 52, 20251 Hamburg, Germany; 2 Center for Population Health Innovation (POINT), University Heart and Vascular Center, Hamburg, Germany; 3 Institute of Molecular Biology (IMB), Mainz, Germany; 4 German Center for Cardiovascular Research (DZHK), Partner Site Hamburg/Kiel/Luebeck, Hamburg, Germany

**Keywords:** Heart failure prevention, Modifiable risk factors, Multidimensional risk assessment, Screening for heart failure

## Abstract

**Purpose of Review:**

Heart failure (HF) is a complex clinical syndrome that develops as the final common manifestation of diverse cardiovascular disorders along a variety of different pathophysiological pathways. The trajectory towards HF is set decades earlier by a combination of non-modifiable risk factors and a substantial number of principally modifiable risk factors. Conceptualizing HF prevention as a continuum — from primordial prevention to tertiary prevention — highlights how consistently these factors determine and drive the risk to develop HF. Early at-risk and pre-HF stages therefore represent the most appropriate window for effective prevention, yet they are still underrated and under-recognized in clinical practice.

**Recent Findings:**

A broad array of modifiable risk factors, together with echocardiographic and laboratory markers, are involved in disease progression. Combining these domains may optimize risk stratification and enable more targeted prevention efforts.

**Summary:**

The rapidly evolving heart failure pandemic, largely driven by the rising prevalence of conditions that are to a substantial extent attributable to modifiable risk factors, poses an enormous burden on health systems worldwide. In consequence, enhanced global awareness, comprehensive assessment and more effective control of modifiable risk factors are urgently needed to prevent HF. A multidimensional risk assessment approach could facilitate early identification and timely intervention to prevent progression to symptomatic HF.

## The Cardiovascular Continuum Through the Heart Failure Lens

Heart failure (HF) is one of the leading causes of cardiovascular morbidity and mortality, affecting over 64 million people worldwide [1]. HF is a heterogeneous clinical syndrome: HF with preserved Ejection Fraction (HFpEF) now accounts for roughly half of newly diagnosed cases, while HF with reduced Ejection Fraction (HFrEF) has declined [2]. HF incidence patterns differ by sex, with women more often developing HFpEF and men more frequently affected by HFrEF [3, 4]. The overall disease burden continues to rise, driven not only by ageing populations but also by increasing prevalence rates of modifiable cardiovascular risk factors such as hypertension, diabetes, and obesity [5]. Preventing HF before the onset of symptoms and structural cardiac abnormalities is therefore crucial to reduce the HF disease burden. Greater focus on risk assessment is essential to enable early identification of at-risk individuals and timely intervention before symptomatic disease develops [6–8].

### Classical Risk Factors and Beyond: Levers for HF prevention

Long-term exposure to cardiovascular risk factors gradually impairs cardiac structure and function, creating a continuum from risk to overt disease.

Risk factors for heart failure can be broadly classified into non-modifiable and modifiable. Non-modifiable risk factors, such as age, sex, and genetic predisposition, primarily determine baseline susceptibility and long-term risk trajectories but are not amenable to intervention. Modifiable risk factors represent the key targets for prevention and comprise both the five traditionally known cardiovascular risk factors and non-traditional risk factors.

Addressing both traditional and non-traditional modifiable risk factors within a comprehensive prevention framework is essential to effectively reduce lifetime heart failure risk.

The five traditional cardiovascular risk factors: smoking, diabetes, unhealthy weight, hypertension and dyslipidemia show a substantial impact along this pathway and markedly increase the risk of clinical cardiovascular events and HF subsequently [9–12].

The *Global Cardiovascular Risk Consortium* recently quantified the impact of these risk factors: smoking, diabetes, systolic blood pressure, non-HDL cholesterol and body mass index account for more than 50% of incident cardiovascular disease and 20% of all-cause mortality [10]. In a follow-up analysis of more than 2 million individuals, those free of these risk factors at the age of 50 years lived more than ten years longer without cardiovascular disease and death than those exposed to all five risk factors [9].

Beyond the five traditional cardiovascular factors, lifestyle behaviors, social and psychosocial determinants may significantly modify HF risk. Regular physical activity and healthy dietary patterns — such as *Dietary Approaches to Stop Hypertension* (DASH diet) or Mediterranean diets — are consistently associated with lower HF incidence, partly mediated by reductions in inflammation, oxidative stress and cardiometabolic disease [13]. Psychosocial stress, depression, social isolation, and adverse childhood experiences, further increase cardiovascular and HF risk through factors such as autonomic imbalance, inflammation and unhealthy coping behaviors [14]. Social determinants of health may independently modulate disease susceptibility by influencing preventive and therapeutic healthcare access, environmental constraints and lifestyle patterns [15].

Because ongoing exposure to these largely modifiable risk factors accelerates progression of cardiac dysfunction and structural changes, early intervention is essential to avert symptomatic HF and recurrent hospitalizations. Targeted prevention and risk factor management can interrupt the development of irreversible cardiac damage before symptomatic HF manifests.

### Risk Assessment: From 10-Year Risk to Lifetime Risk

In addition to modifiable risk factors, pre-existing diseases, comorbidities, and a family history of cardiomyopathies further increase HF risk. Laboratory and imaging markers can also help to identify patients at increased risk in asymptomatic populations, and are essential for targeted prevention [7]. Existing risk prediction models, mainly from the United States, partially already integrate predictors from multiple domains. More recently, artificial intelligence (AI) and machine learning–based approaches have been applied to leverage high-dimensional clinical data, imaging features, and biomarkers. Advanced models incorporating biomarkers such as NT-proBNP and high-sensitivity troponins, as well as AI-driven feature selection, have demonstrated improved risk discrimination [16–19].

Age remains the strongest non-modifiable risk factor for HF. A recent global burden of disease analysis reported that adults over 60 years have an approximately 20-fold higher risk of HF than younger individuals [20], and the lifetime risk of developing HF after age 50 is 25% for men and 23% for women [21]. Although HF often develops later in life, most global risk scores focus on a maximum 10-year risk horizon [16–19].

For younger adults, lifetime risk estimates and “heart age” concepts can make prevention more tangible and support earlier intervention. Serial assessment of modifiable risk factors is essential, and trajectories (e.g. cumulative blood pressure exposure) may be more predictive of HF risk than single measurements [22].

### Pathophysiologic Spectrum and Stages of Heart Failure Development

As HF develops along a biological and clinical spectrum, increasing exposure to cardiovascular risk factors and consecutive clinical conditions occurs long before measurable abnormalities in ejection fraction are detectable. The transition to a manifested clinical syndrome is driven by myocardial stress, inflammation, fibrosis and adverse remodelling of the left ventricle [7].

The Universal Definition of Heart Failure (Table 1), introduced by an international consensus comittee in 2021, defines two asymptomatic stages: Stage A: At risk for HF and Stage B: Pre-HF [6, 7]. Beyond describing the course of the disease, this framework aims to delineate the window in which risk assessment and preventive measures are likely to be most effective.

**Table 1:** Stages of heart failure development

Stage	Description	Key characteristics
Stage A: At risk for HF	Absence of structural or functional abnormalities and no symptoms but presence of conditions strongly associated with HF	Hypertension, diabetes, obesity, atherosclerotic CVD, cardiotoxic agents, genetic variants for or family history of cardiomyopathy
Stage B: Pre-HF	No HF symptoms, but evidence of structural or functional abnormalities or elevated biomarkers of myocardial stress or injury	Structural heart disease, evidence of increased filling pressure, increased natriuretic peptides or cardiac troponins
Stage C: Symptomatic HF	Past or current symptoms/signs caused by structural or functional abnormalities	Dyspnea, fatigue/tiredness, reduced exercise tolerance, peripheral (pitting) edema, elevated jugular venous pressure, pulmonary rales, gallop rhythm
Stage D: Advanced HF	Severe symptoms/signs at rest interfering with life functions and leading to hospitalizations	Refractory, requiring advanced therapies i.e. mechanical circulatory support, transplantation

according to Bozkurt et al. [6] and Heidenreich et al. [24]

Individuals in Stage A comprise more than one-third of the adult population in high-income nations [1]. At this stage, clinical and lifestyle risk factors and comorbidities form the primary basis for risk assessment. In Stage B, however, risk assessment should extend beyond clinical and lifestyle risk factors to include laboratory biomarkers and echocardiography. Since targeted interventions can still prevent or postpone symptomatic heart failure at this stage, identifying Stage B is clinically crucial [6, 23].

A patient enters Stage C when symptoms such as dyspnea, oedema, or exercise intolerance occur. At this stage, HF is further classified as HFrEF, HFmrEF, and HFpEF. Fewer patients progress to Stage D, in which advanced therapies are often required and the focus shifts from prevention to disease management.

### Early Intervention Along the Heart Failure Continuum

**Fig. 1:**
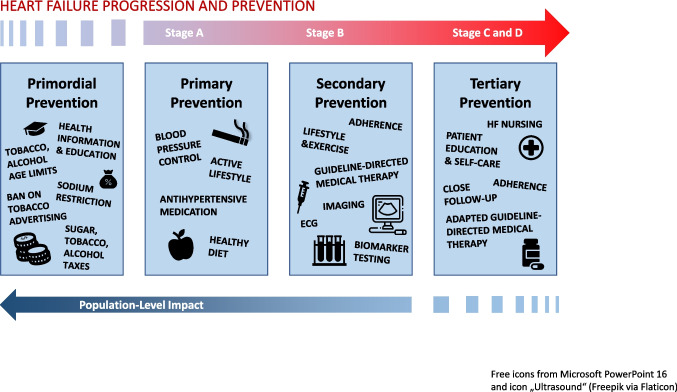
Prevention along the heart failure continuum and stages of heart failure

HF prevention spans the entire disease trajectory (Fig. 1), from cardiovascular risk reduction (primordial and primary prevention) to targeted treatment in individuals with preclinical cardiac abnormalities (secondary prevention) and comprehensive care in symptomatic HF (tertiary prevention). Contemporary guidelines explicitly recognize Stage A (at risk) and Stage B (pre-HF) as most actionable phases for prevention, with a focus on detecting and treating individuals before HF symptoms occur [24].

*Prevention across the heart failure continuum comprises primordial, primary, secondary, and tertiary strategies that evolve with disease progression. Early interventions focus on population-level risk reduction, including health education, lifestyle modification, and blood pressure control, while in advanced stages, prevention focus shifts to individualized, patient-centered care with optimized guideline-directed medical therapy, ongoing diagnostic monitoring, adherence support, patient education, and close follow-up.*

**Primordial prevention** aims to avert the development of cardiovascular risk factors through measures such as health education, tobacco control, sodium reduction, health-promoting urban design (e.g. walk-/bikeable cities), and other fiscal policies (e.g. sugar taxes). The North Karelia Project remains a landmark example, achieving > 80% reductions in CVD mortality through broad policy interventions beginning in the 1980 s [25].

**Primary prevention** targets individuals with established risk factors, corresponding largely to Stage A. Lifestyle modification in high-risk individuals — smoking cessation, increased physical activity, and a healthy diet — is fundamental. In addition, optimal blood-pressure control and lipid-lowering therapy reduces downstream HF risk [26, 27]. SGLT2 inhibitors have emerged as a key option across the cardiometabolic spectrum, reducing first HF events across diverse populations [28, 29].

**Secondary prevention** addresses individuals with subclinical structural or functional cardiac abnormalities, who are at increased risk of progression to symptomatic HF (Stage B). Recent guidelines support natriuretic peptide–based screening, multivariable risk scores, targeted echocardiographic imaging or even AI-enabled electrocardiography to refine risk assessment and trigger early therapy [24, 30]. Timely initiation of guideline-directed drug therapy — ARNI/ACE inhibitors, beta-blockers, MRAs, and SGLT2 inhibitors — forms the therapeutic backbone [30].

Finally, **tertiary prevention** focuses on patients with established, symptomatic heart failure, with the goal of lowering morbidity, preventing hospital readmissions and improving survival and quality of life. Close follow-up examination and adaptation of guideline-directed medical therapy is recommended [30]. Additional agents such as GLP-1/GIP receptor agonists show emerging benefit in HF with obesity [31]. Multidisciplinary HF programs and nurse-led care, supported by telemonitoring and hemodynamic monitoring technologies like CardioMEMS, further improve outcomes [32].

## Conclusions

Preventing HF is less about identifying new targets and more about the consistent, risk-adapted implementation of existing strategies focussing on modifiable risk factors. Future research (Table 2) should prioritise improving early detection, refining multidimensional risk algorithms and evaluating cost-effectiveness of screening strategies combined with risk-based prevention in asymptomatic populations. Integrating HF endpoints into global risk discussions, adopting lifetime risk metrics, and aligning cardiology, primary care and public health efforts are key steps towards a future with fewer individuals crossing the threshold into symptomatic HF.

**Table 2:** Urgent research topics and gaps by domain

Domain	Research Topics and Gaps
Risk assessment and prediction algorithms	Development and population-specific validation of multidimensional risk algorithms integrating clinical and lifestyle factors, biomarkers, imaging, comorbidities and social determinants, improve precision, equity, and clinical usability of risk tools
Screening and early detection	Improving population-based or risk-adapted screening strategies (populations at risk, timing, intervals, modalities), linked to preventive care pathways, and economic evaluation including long-term outcomes
Implementation science	Translating preventive strategies into real-world practice by improving awareness and uptake, adherence, persistence, and coordination across care settings
Risk factor modification and lifetime trajectories	Investigating the impact of early and sustained modification of modifiable risk factors on lifetime heart failure risk and disease trajectories

## Key References

- Khan SS, Breathett K, Braun LT, Chow SL, Gupta DK, Lekavich C, et al. Risk-Based Primary Prevention of Heart Failure: A Scientific Statement From the American Heart Association. Circulation. 2025;151(20):e1006-e26.
  - Outlines a risk-based framework for primary prevention of heart failure, emphasizing opportunities for early identification and implementation of preventive strategies in at-risk but asymptomatic populations.
- Magnussen C, Ojeda FM, Leong DP, Alegre-Diaz J, Amouyel P, Aviles-Santa L, et al. Global Effect of Modifiable Risk Factors on Cardiovascular Disease and Mortality. N Engl J Med. 2023;389(14):1273-85.
  - Quantifies the global contribution of modifiable risk factors to cardiovascular disease and mortality, underscoring the major preventive potential of risk factor control across populations.
- Bozkurt B, Coats AJS, Tsutsui H, Abdelhamid CM, Adamopoulos S, Albert N, et al. Universal definition and classification of heart failure: a report of the Heart Failure Society of America, Heart Failure Association of the European Society of Cardiology, Japanese Heart Failure Society and Writing Committee of the Universal Definition of Heart Failure: Endorsed by the Canadian Heart Failure Society, Heart Failure Association of India, Cardiac Society of Australia and New Zealand, and Chinese Heart Failure Association. Eur J Heart Fail. 2021;23(3):352-80.
  - Provides a universal definition and classification system for heart failure, improving consistency in diagnosis, staging, and identification of patients in pre-heart failure phases, also serving as a conceptional basis for prevention.

## Data Availability

No datasets were generated or analysed during the current study.

## References

[1] Savarese G, Becher PM, Lund LH, Seferovic P, Rosano GMC, Coats AJS. Global burden of heart failure: a comprehensive and updated review of epidemiology. Cardiovasc Res. 2022;118(17):3272–87. 10.1093/cvr/cvac013 35150240

[2] Desai N, Olewinska E, Famulska A, Remuzat C, Francois C, Folkerts K. Heart failure with mildly reduced and preserved ejection fraction: a review of disease burden and remaining unmet medical needs within a new treatment landscape. Heart Fail Rev. 2024;29(3):631–62. 38411769 10.1007/s10741-024-10385-y PMC11035416

[3] Shah KS, Xu H, Matsouaka RA, Bhatt DL, Heidenreich PA, Hernandez AF, et al. Heart Failure With Preserved, Borderline, and Reduced Ejection Fraction: 5-Year Outcomes. J Am Coll Cardiol. 2017;70(20):2476–86. 29141781 10.1016/j.jacc.2017.08.074

[4] Delco A, Portmann A, Mikail N, Rossi A, Haider A, Bengs S, et al. Impact of sex and gender on heart failure. Cardiovasc Med. 2023;26(3):88.

[5] Groenewegen A, Rutten FH, Mosterd A, Hoes AW. Epidemiology of heart failure. Eur J Heart Fail. 2020;22(8):1342–56. 32483830 10.1002/ejhf.1858 PMC7540043

[6] Bozkurt B, Coats AJS, Tsutsui H, Abdelhamid CM, Adamopoulos S, Albert N, et al. Universal definition and classification of heart failure: a report of the Heart Failure Society of America, Heart Failure Association of the European Society of Cardiology, Japanese Heart Failure Society and Writing Committee of the Universal Definition of Heart Failure: endorsed by the Canadian Heart Failure Society, Heart Failure Association of India, Cardiac Society of Australia and New Zealand, and Chinese Heart Failure Association. Eur J Heart Fail. 2021;23(3):352–80. 33605000 10.1002/ejhf.2115

[7] Lala A, Beavers C, Blumer V, Brewer L, De Oliveira-Gomes D, Dunbar SB, et al. The continuum of prevention and heart failure in cardiovascular medicine: a joint scientific statement from the Heart Failure Society of America and The American Society for Preventive Cardiology. J Card Fail. 2025. 10.1016/j.ajpc.2025.101069.41381127

[8] Khan SS, Breathett K, Braun LT, Chow SL, Gupta DK, Lekavich C, et al. Risk-based primary prevention of heart failure: a scientific statement from the American Heart Association. Circulation. 2025;151(20):e1006–26. 40235437 10.1161/CIR.0000000000001307

[9] Magnussen C, Alegre-Diaz J, Al-Nasser LA, Amouyel P, Aviles-Santa L, Bakker SJL, et al. Global Effect of Cardiovascular Risk Factors on Lifetime Estimates. N Engl J Med. 2025;393(2):125–38. 40162648 10.1056/NEJMoa2415879 PMC13498409

[10] Magnussen C, Ojeda FM, Leong DP, Alegre-Diaz J, Amouyel P, Aviles-Santa L, et al. Global Effect of Modifiable Risk Factors on Cardiovascular Disease and Mortality. N Engl J Med. 2023;389(14):1273–85. 37632466 10.1056/NEJMoa2206916 PMC10589462

[11] Kamimura D, Cain LR, Mentz RJ, White WB, Blaha MJ, DeFilippis AP, et al. Cigarette smoking and incident heart failure: insights from the Jackson Heart Study. Circulation. 2018;137(24):2572–82. 29661945 10.1161/CIRCULATIONAHA.117.031912 PMC6085757

[12] Lu Y, Xu Z, Georgakis MK, Wang Z, Lin H, Zheng L. Smoking and heart failure: a Mendelian randomization and mediation analysis. ESC Heart Fail. 2021;8(3):1954–65. 33656795 10.1002/ehf2.13248 PMC8120408

[13] Zhu Z, Li FR, Jia Y, Li Y, Guo D, Chen J, et al. Association of lifestyle with incidence of heart failure according to metabolic and genetic risk status: a population-based prospective study. Circ Heart Fail. 2022;15(9):e009592. 35975661 10.1161/CIRCHEARTFAILURE.122.009592

[14] Carola V, Vincenzo C, Di Vincenzo G, Morale C, Cecchi V, Nicolais G. Psychological risk factors and cardiovascular disease. Front Psychol. 2024;15:1419731. 39403242 10.3389/fpsyg.2024.1419731 PMC11471649

[15] Bazoukis G, Loscalzo J, Hall JL, Bollepalli SC, Singh JP, Armoundas AA. Impact of social determinants of health on cardiovascular disease. J Am Heart Assoc. 2025;14(5):e039031. 40035388 10.1161/JAHA.124.039031 PMC12132660

[16] Agarwal SK, Chambless LE, Ballantyne CM, Astor B, Bertoni AG, Chang PP, et al. Prediction of incident heart failure in general practice: the Atherosclerosis Risk in Communities (ARIC) Study. Circ Heart Fail. 2012;5(4):422–9. 22589298 10.1161/CIRCHEARTFAILURE.111.964841 PMC3412686

[17] Nambi V, Liu X, Chambless LE, de Lemos JA, Virani SS, Agarwal S. Troponin T and N-terminal pro–B-type natriuretic peptide: a biomarker approach to predict heart failure risk—the Atherosclerosis Risk in Communities Study. Clin Chem. 2013;59(12):1802–10. 24036936 10.1373/clinchem.2013.203638 PMC4208068

[18] Seliger SL, Hong SN, Christenson RH, Kronmal R, Daniels LB, Lima JAC. High-sensitive cardiac troponin T as an early biochemical signature for clinical and subclinical heart failure: MESA (Multi-Ethnic Study of Atherosclerosis). Circulation. 2017;135(16):1494–505. 28159799 10.1161/CIRCULATIONAHA.116.025505 PMC5401621

[19] Khan SS, Ning H, Shah SJ, Yancy CW, Carnethon M, Berry JD. 10-year risk equations for incident heart failure in the general population. J Am Coll Cardiol. 2019;73(19):2388–97. 31097157 10.1016/j.jacc.2019.02.057 PMC6527121

[20] Ran J, Zhou P, Wang J, Zhao X, Huang Y, Zhou Q, et al. Global, regional, and national burden of heart failure and its underlying causes, 1990-2021: results from the global burden of disease study 2021. Biomark Res. 2025;13(1):16. 39849627 10.1186/s40364-025-00728-8 PMC11755835

[21] Vasan RS, Enserro DM, Beiser AS, Xanthakis V. Lifetime risk of heart failure among participants in the Framingham Study. J Am Coll Cardiol. 2022;79(3):250–63. 35057911 10.1016/j.jacc.2021.10.043 PMC8820638

[22] Pool LR, Ning H, Wilkins J, Lloyd-Jones DM, Allen NB. Use of long-term cumulative blood pressure in cardiovascular risk prediction models. JAMA Cardiol. 2018;3(11):1096–100. 30193291 10.1001/jamacardio.2018.2763 PMC6583053

[23] McDonagh TA, Metra M, Adamo M, Gardner RS, Baumbach A, Böhm M, et al. 2021 ESC Guidelines for the diagnosis and treatment of acute and chronic heart failure. Eur Heart J. 2021;42(36):3599–726. 34447992 10.1093/eurheartj/ehab368

[24] Heidenreich P, Bozkurt B, Aguilar D, Allen LA, Byun JJ, Colvin MM, et al. 2022 AHA/ACC/HFSA Guideline for the Management of Heart Failure: Executive Summary: A Report of the American College of Cardiology/American Heart Association Joint Committee on Clinical Practice Guidelines. Circulation. 2022;145(18):e876–94. 35363500 10.1161/CIR.0000000000001062

[25] Puska P, Jaini P. The North Karelia project: prevention of cardiovascular disease in Finland through population-based lifestyle interventions. Am J Lifestyle Med. 2020;14(5):495–9. 32922234 10.1177/1559827620910981 PMC7444010

[26] Wright JT Jr, Williamson JD, Whelton PK, Snyder JK, Sink KM, Rocco MV, et al. A Randomized Trial of Intensive versus Standard Blood-Pressure Control. N Engl J Med. 2015;373(22):2103–16. 26551272 10.1056/NEJMoa1511939 PMC4689591

[27] Rethy L, Cohen JB. Intensive blood pressure lowering for prevention of heart failure with preserved and reduced ejection fractions. Circ Heart Fail. 2021;14(12):e009277. 34823370 10.1161/CIRCHEARTFAILURE.121.009277 PMC8692331

[28] Zelniker TA, Wiviott SD, Raz I, Im K, Goodrich EL, Bonaca MP, et al. SGLT2 inhibitors for primary and secondary prevention of cardiovascular and renal outcomes in type 2 diabetes: a systematic review and meta-analysis of cardiovascular outcome trials. Lancet. 2019;393(10166):31–9. 30424892 10.1016/S0140-6736(18)32590-X

[29] Usman MS, Siddiqi TJ, Anker SD, Bakris GL, Bhatt DL, Filippatos G, et al. Effect of SGLT2 inhibitors on cardiovascular outcomes across various patient populations. J Am Coll Cardiol. 2023;81(25):2377–87. 37344038 10.1016/j.jacc.2023.04.034

[30] McDonagh TA, Metra M, Adamo M, Gardner RS, Baumbach A, Böhm M, et al. 2023 focused update of the 2021 ESC guidelines for the diagnosis and treatment of acute and chronic heart failure. Eur Heart J. 2023;44(37):3627–39. 37622666 10.1093/eurheartj/ehad195

[31] Packer M, Zile MR, Kramer CM, Baum SJ, Litwin SE, Menon V, et al. Tirzepatide for heart failure with preserved ejection fraction and obesity. N Engl J Med. 2025;392(5):427–37. 39555826 10.1056/NEJMoa2410027

[32] Angermann CE, Assmus B, Anker SD, Asselbergs FW, Brachmann J, Brett ME, et al. Pulmonary artery pressure-guided therapy in ambulatory patients with symptomatic heart failure: the CardioMEMS European Monitoring Study for Heart Failure (MEMS-HF). Eur J Heart Fail. 2020;22(10):1891–901. 32592227 10.1002/ejhf.1943

